# Aryl Hydrocarbon Receptor: From Homeostasis to Tumor Progression

**DOI:** 10.3389/fcell.2022.884004

**Published:** 2022-04-07

**Authors:** Claudia Rejano-Gordillo, Ana Ordiales-Talavero, Ana Nacarino-Palma, Jaime M. Merino, Francisco J. González-Rico, Pedro M. Fernández-Salguero

**Affiliations:** ^1^ Departamento de Bioquímica y Biología Molecular y Genética, Facultad de Ciencias, Universidad de Extremadura, Badajoz, Spain; ^2^ Chronic Diseases Research Centre (CEDOC), Rua Do Instituto Bacteriológico, Lisboa, Portugal

**Keywords:** aryl hydrocarbon receptor, differentiation, pluripotency, reprogramming, chromatin

## Abstract

Transcription factor aryl hydrocarbon receptor (AHR) has emerged as one of the main regulators involved both in different homeostatic cell functions and tumor progression. Being a member of the family of basic-helix-loop-helix (bHLH) transcriptional regulators, this intracellular receptor has become a key member in differentiation, pluripotency, chromatin dynamics and cell reprogramming processes, with plenty of new targets identified in the last decade. Besides this role in tissue homeostasis, one enthralling feature of AHR is its capacity of acting as an oncogene or tumor suppressor depending on the specific organ, tissue and cell type. Together with its well-known modulation of cell adhesion and migration in a cell-type specific manner in epithelial-mesenchymal transition (EMT), this duality has also contributed to the arise of its clinical interest, highlighting a new potential as therapeutic tool, diagnosis and prognosis marker. Therefore, a deregulation of AHR-controlled pathways may have a causal role in contributing to physiological and homeostatic failures, tumor progression and dissemination. With that firmly in mind, this review will address the remarkable capability of AHR to exert a different function influenced by the phenotype of the target cell and its potential consequences.

## Introduction

The intracellular dioxin receptor (AHR) has distinctive functional and structural properties among the family of basic-helix-loop-helix (bHLH) transcriptional regulators (Roman et al., 2018). Initially discovered as a receptor to a variety of xenobiotics compounds, the signaling pathways leading to AHR activation by exogenous ligands, such as 2,3,7,8-Tetrachlorodibenzodioxin (TCDD), has been extensively studied. The non-activated form of AHR is located in the cytoplasm in a complex with several chaperones, among which are two HSP90 (Heat Shock Protein 90), a co-chaperone p23, a XAP-molecule 2 (hepatitis B Virus X-associated protein 2) (Larigot et al., 2018). Upon ligand binding, the receptor translocates to the nucleus and heterodimerizes with the class II bHLH protein ARNT/HIF1β (Aryl hydrocarbon receptor nuclear translocator/Hypoxia-inducible factor 1β) (Reyes et al., 1992; (Mulero-Navarro and Fernandez-Salguero, 2016). After transcriptional regulation, the AHR-ARNT heterodimer is disassembled from DNA and AHR is driven again to the cytosol for proteosomal degradation (Davarinos and Pollenz, 1999; Ma and Baldwin, 2000; Santiago-Josefat et al., 2001). Interestingly, the early presence of AHR in metazoans, its high degree of conservation among species and the altered phenotypes observed in several organs, including the liver, in AHR^−/−^ mice (Pohjanvirta et al., 2012) demonstrated its role in tissue homeostasis. Genome-wide and cell signaling studies have shown that lack of AHR significantly alters gene expression in both normal liver (Tij et al., 2006; Moreno-Marín et al., 2018) and hepatoma cells (Sartor et al., 2009). One intriguing feature of AHR is that its functions depend on the phenotype of the target cell, acting as a tumor suppressor or as an oncogene upon specific cell types, tissues or organs (Marlowe and Puga, 2005; Barouki et al., 2007). Furthermore, AHR has a role in reprogramming and in adjusting the rate of organ regeneration after injury. In addition, few studies have suggested that AHR may have a role in senescence since it seems to attenuate lung parenchyma inflammation by controlling senescence (Guerrina et al., 2018). Moreover, human keratinocytes exposed to the AHR ligand TCDD become immortalized by repressing p16 and p53 (Ray and Swanson, 2004).

For certain organs such as the liver, physiological terminal differentiation and proliferation exhaust of hepatocytes is essential for its functionality (Shiojiri et al., 1991; Gentric et al., 2012; Schoenfelder and Fox, 2015). From a functional perspective, the adult (differentiated) liver increases the size of hepatocytes, amplifies gene expression profiles, adjusts its metabolism (Zielke et al., 2013; Schoenfelder and Fox, 2015) and, importantly, gains regenerative capacity upon injury. After exposure to damaging agents or following partial hepatectomy, liver stem cells or primary hepatocytes enter cell cycle to regenerate the injured tissue (Taub, 2004; Forbes and Newsome, 2016; Yagi et al., 2020). Several works have also identified the reprogramming and pluripotency factors OCT4-KLF4-SOX2-MYC (OKSM) as key in the progression of different tumors, including hepatocarcinoma (Wang and Herlyn, 2015; Kuo et al., 2016; Zhou et al., 2016). Remarkably, cell reprogramming appears closely linked to senescence, a seemingly opposed cell status that represents a hallmark of aging in response to various stress stimuli (López-Otín et al., 2013; Chiche et al., 2020). Indeed, recent observations support that tissue injury induces senescence and activates signaling pathways controlling reprogramming, thus highlighting the functional association of both processes (Mosteiro et al., 2016a; Chiche et al., 2017; Mosteiro et al., 2018). The reprogramming-senescence axis thus have a major role in normal development and tissue regeneration and remodeling in response to damage (Rhinn et al., 2019). Consequently, AHR has been described as a tumor suppressor or an oncogene, depending on the types of cancer and study cohorts in the same type of cancer (Sun, 2021). Moreover, hepatocellular carcinoma is the most malignant and with worse prognosis liver tumor with an increasing worldwide incidence (Kim et al., 2014). Most patients are diagnosed at advanced stages of the disease when therapeutic opportunities are very limited (Llovet et al., 2016). With the multikinase inhibitor Sorafenib providing a poor increase in overall survival (Llovet et al., 2008), it is therefore crucial to identify and characterize novel prognostic markers and more efficient and specific therapeutic strategies.

Altogether, this review covers the main aspects of the AHR role in tissue repair and reprogramming likely through the control of signaling pathways in differentiation, pluripotency and senescence.

## Involvement of Aryl Hydrocarbon Receptor in Tissue Homeostasis and Regeneration

Since AHR possess an important implication in different physiological processes, alterations in its signaling pathway can lead to homeostatic disorders, covering from development, differentiation, pluripotence, proliferation, regeneration, tumor progression and senescence. Those disorders can affect a variety of organs such as liver, lung, skin and brain. A crucial regulator of cell proliferation, viability and ploidy is the signaling network driven by the insulin receptor (INS-R) and downstream PI3K (phosphatidylinositol-3-phosphate kinase) pathway (Celton-Morizur et al., 2010; Cui and Yu, 2016). It has been recently described that the lack of AHR increases the activation of the phospho-IRS-2 substrate, a major INS-R intermediate protein in the liver (Moreno-Marín et al., 2018). Also, in AHR-null mice the interaction and expression levels of phospho-IRS-2 and PI3K were increased when compared to wild type mice (Moreno-Marín et al., 2018). It is also known that Serine-threonine protein kinase-B/AKT (onwards AKT) is the required PI3K signaling intermediate in most cell types (Cui and Yu, 2016). Furthermore, the active phospho-AKT (p-AKTSer473) form presented an upregulation in AHR knockout mice (Moreno-Marín et al., 2018). Both AKT phosphorylation and PI3K activity are negatively regulated by the phosphatase and tensin homolog (PTEN) (Bunney and Katan, 2010). Consequently, the lack of AHR promoted a PTEN downregulation with an inverse pattern versus phospo-AKT. For those reasons, there is a clear association established between the lack of AHR, proliferation and a sustained overactivation of the INS-R/PI3K pathway (Moreno-Marín et al., 2018).

The PI3K signaling is also largely known for the inhibition the p53 tumor suppressor to block apoptosis in proliferating cells (Sabbatini and McCormick, 1999; Yamaguchi et al., 2001), with recent studies showing that p53 has relevant functions in preventing polyploidy in mature cells (Aylon and Oren, 2011; Kurinna et al., 2013). Regarding that regulation, the p21Cip1 protein (p21Cip1), a relevant p53 target, is also involved in repressing cell proliferation (Jung et al., 2010; Karimian et al., 2016). The axis between PI3K and AKT is also related to Wnt/β-Cat signaling via downstream target GSK3β, a component of the Wnt/β-Cat degradation complex (Nusse and Clevers, 2017).

**FIGURE 1:**
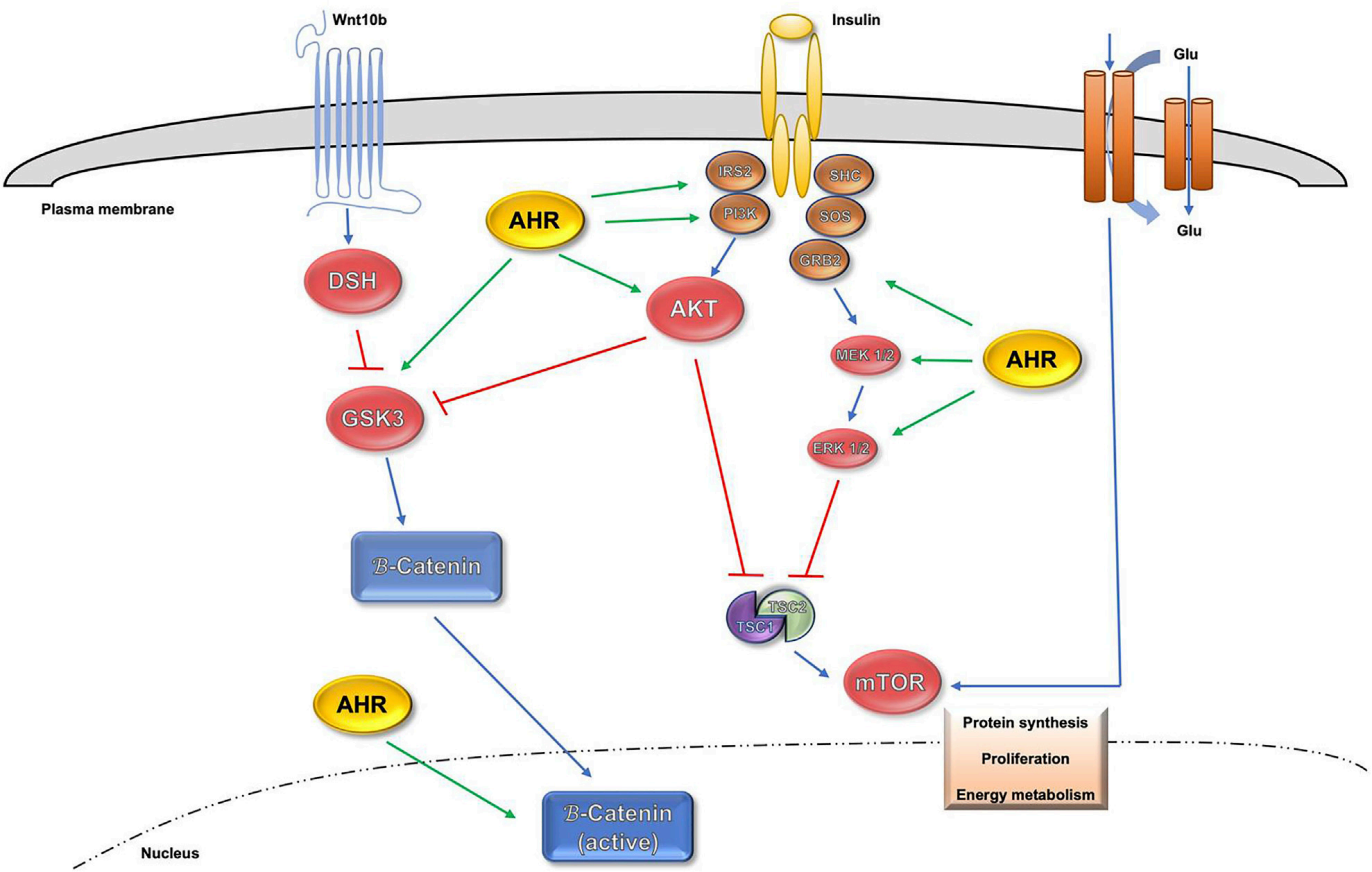
Influence of AHR in AKT, mTOR and β-Catenin signaling pathways.

One thrilling aspect was the discovery of the simultaneous participation of the mammalian target of rapamycin (mTOR) in several signaling pathways controlling metabolism, cell differentiation and proliferation, with special relevance of those mediated by PI3K, ERK and Wnt/β-Cat, which activate the mTORC1 complex through the guanosine triphosphate (GTP)-binding protein RHEB (Laplante and Sabatini, 2009; Laplante and Sabatini, 2012; Saxton and Sabatini, 2017). Furthermore, the ribosomal S6 kinase-1 (S6K1), a major target of the mTORC1 complex, is activated by phosphorylation (Laplante and Sabatini, 2009; Laplante and Sabatini, 2012; Saxton and Sabatini, 2017), but also implicated in the control of polyploidy (Ma et al., 2009). In this regard, the activation of INS-R/PI3K/ERK and Wnt/β-Cat signaling pathways that takes place during liver maturation in AHR^−/−^ mice maintains proliferation and inhibits differentiation-related polyploidy by assembling the mTORC1 complex (Moreno-Marín et al., 2018). Moreover, the use of the pharmacological inhibitors salinomycin (Wnt/β-Cat), LY294002 (PI3K) and PD98059 (ERK) resulted in a partial rescue of polyploidy in AHR-null mice liver (Moreno-Marín et al., 2018). Besides, AHR acts like a greater regulator of signalling pathways positively related to stemness such as the hippo-YAP pathway and the Wnt-βcatenin pathway (Procházková et al., 2011; Moreno-Marín et al., 2017). The interplay involving AHR and those signaling pathways can be seen in Figure 1.

### Aryl Hydrocarbon Receptor in Regenerative Processes

The fact that tissue regeneration is a necessary process to maintain tissular homeostasis connects with some of them having high rates of regeneration through life under normal physiological conditions. However, this ability has a great importance to replace body parts after injuries and/or pathological processes of different kinds, which can damage the organs and cause a loss of mass. This capacity differs between species, and even in the tissues of the same organism (Goldman and Poss, 2020). In no-mammalian species is so effective that it can be regenerated the whole organisms from small body fragments (Poss, 2010), while in mammalian species, tissue regeneration is restricted to only some organs, including skeletal muscle, liver, intestinal epithelium, skin and blood (Rafii et al., 2016; Mokalled and Poss, 2018; Wells and Watt, 2018; Wosczyna and Rando, 2018). To achieve the regenerative process, a great number of molecular pathways must orchestrate the determination of regenerative capacity; the balance between stem cells, dedifferentiation and transdifferentiation; how regenerative signals are initiated and targeted; and the mechanisms that control proliferation cellular and patterning

In regenerative processes, it has been demonstrated that some transcription factors (TF) can control cell identities and different cellular responses. In particular, the introduction of 4TFs (OCT4, SOX2, KLF4 and MYC) gives the necessary capacitation to revert differentiated fibroblasts into pluripotent stem cells, providing them with similar characteristics to embryonic stem cells (Takahashi and Yamanaka, 2006). AHR has been presented as another TF involved in a variety of physiologic functions (Fernandez-Salguero et al., 1995; Fernandez-Salguero et al., 1997; Mulero-Navarro and Fernandez-Salguero, 2016) that has been recently related with those Yamanaka factors in the regulation of pluripotency and differentiation state in early mouse embryogenesis (Nacarino-Palma et al., 2021a) and other differentiation and pluripotency processes (Morales-Hernández et al., 2016; González-Rico et al., 2020; Rico-Leo et al., 2021). Several studies evidence that the activation of the genetic programs involved in embryogenesis are both critical and dominant in regeneration (Fausett and Goldman, 2006; Lepilina et al., 2006; Ransom et al., 2018). In this way, it has been shown that AHR is involved in the regulation of pluripotency markers OCT4 and NANOG in organs like the lung (Morales-Hernández et al., 2017) and the liver (Moreno-Marín et al., 2017), but also in the study of regeneration models after acute toxic damage in rodents and different cell lines (Ko et al., 2016; Ko and Puga, 2017). In addition, recent studies have associated activation of these pluripotency factors with a stem-like phenotype (Wagner et al., 2010; Cheung et al., 2011; Safa, 2016). Furthermore, SOX2 and KLF4 has been found to be critical in a stem cell population located in the olfactory epithelium and retinal ganglion neurons during the regenerative process (Gadye et al., 2017; Rocha-Martins et al., 2019). In a similar manner, AHR also affects stemness capacity in different environments; its activation impairs bone-marrow-derived stem cells differentiation into osteoblasts (Korkalainen et al., 2009). The lack of the receptor in AHR-null mice increases the stem population in repairing lung and liver (Morales-Hernández et al., 2017; Moreno-Marín et al., 2017); while AHR activation in hematopoietic stem/progenitor cells affects cellular proliferation, trafficking and migration (Sakai et al., 2003; Casado et al., 2011; Singh et al., 2011). In the KrasG12D-AHR–/– mouse model, lungs contain increased numbers of cells expressing markers for both progenitor clara and alveolar type II cells, and also have elevated numbers of cells positive for pluripotent stem cells markers (Nacarino-Palma et al., 2021b).

However, extensively proliferating stem cell and non-stem cell populations are required to ensure restoration of damaged tissue. In the last decades, different studies have shown that AHR group II targets include genes involved in the control of proliferation, pointing out the Ah receptor participation as a modulator of the cell cycle through the regulation of G1/S phase progression. The compound TCDD can impair liver regeneration after two-thirds partial hepatectomy (PHx) by controlling the levels of the cyclin kinase inhibitors p21Cip1 and p27Kip1 (Jackson et al., 2014). Similarly, different treatments with AHR ligands can trigger its sustained activation, causing cell cycle arrest in G1 in 5 L (Wiebel et al., 1991; Reiners et al., 1999; Santini et al., 2001), Hepa-1c1c7 (Marlowe et al., 2004) and MCF7 cell lines (Trapani et al., 2003). The accumulation of the AHR transcriptional target, *Cyp1a1*, works as a negative feedback mechanism to eliminate endogenous AHR ligands ensuring correct cell proliferation (Levine-Fridman et al., 2004). Other studies have shown that AHR forms complexes with the RB protein (Ge and Elferink, 1998; Puga et al., 2000; Chan et al., 2001), acting as a negative regulator of cell cycle progression by inhibiting the dependent transcriptional activity by E2F.

The initiation of the cell cycle may be related to acute inflammation mediated by the innate immune system. The AHR relevance in the regulation of the immune system is strongly emerging, as shown by recent studies that describe the limitation of macrophage responses to inflammatory stimuli dependent on AHR activation (Gutiérrez-Vázquez and Quintana, 2018). Modulation of AHR activation can potentially redirect the immune cells toward an antitumoral phenotype, therefore representing a novel therapeutic approach in immuno-oncology (Sun, 2021). The formation of AHR-RelA complexes may also help explain some of the adverse toxicological outcomes of AHR ligands such as immunosuppression, thymic involution, hyperkeratosis, and carcinogenesis (Marlowe and Puga, 2005). Within the same family, RelB exerts a role in the regulation of genes activated in an AHR-dependent manner such as cytokines IL-17A, IL-22 in both bone marrow-derived macrophages (BMM) and thymus, with TCDD inducing also IDO1/IDO2 expression only in thymus (Ishihara et al., 2019). Such cross-talk between AhR and NF-kB pathways has also been found to regulate AhR-mediated gene transcription of IL6 and IL8 in breast cancers (Tian et al., 1999; Vogel et al., 2007). On the other hand, AHR activation by TCDD in human osteosarcoma cells is associated with an increased aggressiveness, leading to a higher expression level of receptor activator of NF-kB ligand (RANKL) (Yang et al., 2018). Moreover, it has been reported that AHR can bind to tumor suppressor KLF6, unmasking a novel AhR signalling mechanism distinct from the canonical XRE-driven process (Wilson et al., 2013). In addition, the AHR/NF-kB axis is able to modulate Pb (lead)-induced toxicity in human lung cancer cells (Attafi et al., 2020).

The rising of many studies in different mammalian models supports the direct involvement of AHR in cell regeneration by modulating different signalling pathways essential in this process. AHR activation inhibits regenerative hepatocyte growth following partial hepatectomy, resulting in p21^Cip1^ increased expression in mice (Mitchell et al., 2006; Jackson et al., 2014) and AHR-mediated regulation of cell cycle progression in hepatectomized rats (Bauman et al., 1995). AHR-null mice improves the lungs and liver regeneration after exposition to acute toxic compounds through the increase in stem-like cells population (Morales-Hernández et al., 2017; Moreno-Marín et al., 2017). Also, AHR have a main implication in bone diseases, particularly in the role of environmental pollutants that induce bone loss. Regarding that, AHR participates in bone remodelling through altering the interplay between bone-forming osteoblasts and bone-resorbing osteoclasts in human osteosarcoma cells (Park et al., 2020); also inhibits osteogenic differentiation in human Osteoblast-Like Cells (Yun et al., 2018); and its inhibition leads to an increase in bone mineral density (BMD) and bone strength in murine models (Yu T.-Y. et al., 2014).

Regarding the use of non-mammalian animal models like zebrafish, AHR has been confirmed to have a causal role in regeneration. AHR activation impairs heart regeneration in adult zebrafish reducing dysregulated expression of genes involved in heart function, tissue regeneration, cell growth, and extracellular matrix (Hofsteen et al., 2013). Furthermore, AHR has been presented as a crucial regulator of restorative neurogenesis in the zebrafish brain, controlling ependymoglia differentiation towards post-mitotic neurons (Di Giaimo et al., 2018). Finally, AHR activation by TCCD inhibits zebrafish fin regeneration, with recent genomic analysis revealing a functional cross talk between AHR and the well-established Wnt/β-catenin signal transduction pathway (Zodrow and Tanguay, 2003; Mathew et al., 2006; Andreasen et al., 2007).

All these studies suggest that targeting AHR to promote tissue regeneration could be a useful strategy to avoid disturbances of homeostasis that can promote disease, providing a biological foundation for potential regenerative medicine approaches.

## Aryl Hydrocarbon Receptor Role in Pluripotency and Differentiation

The AHR role in cell differentiation has been intensively studied during the last decades. Preliminary studies with HL60 and HEL cell lines showed that the differentiation from monocytes to macrophages with phorbol esters required the transcriptional activation of AHR (Hayashi et al., 1995). Moreover, experiments performed to differentiate AHR +/+ and AHR −/− mouse embryonic fibroblasts (MEFs) to adipocytes revealed that AHR deficiency impairs the differentiation process, suggesting that AHR could be an early regulator of adipogenesis (Alexander et al., 1998). Moreover, the accumulation of TCDD in adipose tissue induces an effect on oxidative stress enzymes in both adipocytes and liver, exacerbating oxidative stress (Kern et al., 2002). Furthermore, TCCD activation of AHR in conjunction with MEK/ERK inhibits the peroxisome proliferator-activated receptor (PPARγ1), leading to a suppression of adipogenesis (Cimafranca et al., 2004). On the other hand, the administration of the AHR exogenous ligand TCDD in pregnant female rats accelerated the differentiation process during the organogenesis of the embryo (Blankenship et al., 1993), suggesting the AHR role in promoting *in vivo* differentiation.

Regarding mouse embryonic development, recent studies showed that the activation of AHR by exogenous ligand in blocks the ability of hematopoietic stem cells for long-term self-renewal (Laiosa et al., 2016). Furthermore, sustained AHR activation during early differentiation of mouse embryonic stem cells compromises critical signaling for cardiac mesoderm ontogeny and cardiomyocyte functions (Wang et al., 2016), indicating that the receptor has a relevant function in cell differentiation inherent in the development of the organism. Moreover, AHR has a relevant role in the early stages of embryonic stem cell differentiation, regulating the core pluripotency network of transcription factors OCT4/POU5F1, NANOG, and SOX2 at initial developmental stages. The lack of AHR in early mouse embryos generates a delay in the expression of such differentiation markers, resulting in a more pluripotent state of AHR-null embryos (Nacarino-Palma et al., 2021a). Also, other studies have shown that AHR promotes the differentiation of human embryoid teratoma cells through inhibition of OCT4 and NANOG expression (Morales-Hernández et al., 2016; González-Rico et al., 2020). A new molecular mechanism was discovered involving Alu retrotransposable elements located in the promoters of pluripotency genes OCT4 and NANOG, containing AHR binding sites, where the Alu-derived transcripts are processed through the miRNA pathway to generate small noncoding RNAs, complementary to the 3′UTR region of NANOG and OCT4. This complementarity reduces the mRNA levels of pluripotency genes, exerting the repressive process (Morales-Hernández et al., 2016). Furthermore, the absence of receptor in mice causes an undifferentiated phenotype in numerous tissues due to the overexpression of pluripotency genes and the accumulation of stem cells subpopulations, originating a regenerative advantage (Morales-Hernández et al., 2016; Morales-Hernández et al., 2017; Moreno-Marín et al., 2017). In that context, AHR-null mice developed a faster and more efficient repair of the lung bronchiolar epithelium upon non-AHR-ligand toxic molecule naphthalene injury. The AHR absence originates an earlier and more efficient activation of stem-like cell subpopulations, besides AHR acts as a modulator of the expression of pluripotency-inducing factors, which are being positively regulated upon lack of AHR. This AHR deficiency improves the regenerative potential in response to the effects of acute toxin exposure (Morales-Hernández et al., 2017). These results contribute to the strong current interest in regenerative medicine to develop modulators to improve tissue repair requiring increased cell proliferation and the earlier activation of progenitor populations.

Furthermore, whole-genome analysis of chromatin immunoprecipitation assays of hepatocellular carcinoma cells from wild-type and AHR knock-out mice allowed the identification of several groups of genes involved in cell differentiation and development directly regulated by AHR (Sartor et al., 2009), together with several studies showing that AHR is necessary for the proper differentiation of lymphocytes by mechanisms that are both dependent and independent of their binding to XRE elements (Quintana et al., 2008; Esser et al., 2009; Veldhoen et al., 2009; Mezrich et al., 2010). Besides, AHR has a crucial role in the differentiation of neuroblastoma cells *in vivo*, maintaining an inverse correlation with the prognostic marker MYCN (Wu et al., 2014). In HL60 human leukemia cells, AHR levels increase during cell differentiation, with classic stem cell marker OCT4 expression decreased, indicating that positive regulation of AHR in leukemia cells could favor a cell differentiated phenotype (Ibabao et al., 2015).

In fact, comparative transcriptomic analysis of keratinocytes of AHR +/+ and AHR −/− mice showed a reduction in the expression of differentiation genes in the AHR-null model (van den Bogaard et al., 2015), while treatment of mouse primary keratinocytes with AHR antagonists CH223191 and GNF351 compromised their terminal differentiation. Interestingly, it has been shown that AHR cooperates with the inducible hypoxia factor HIF-1α in the differentiation of regulatory T cells type 1 (Tr1) through their metabolic reprogramming (Mascanfroni et al., 2015).

Together, these studies have uncovered the involvement of AHR in the differentiation process of several organs like the skin, the intestinal epithelium, the lung epithelium and even the immune system (Esser and Rannug, 2015). Although AHR acts as a differentiating factor in most of the studied cell types, its activation by TCDD can also inhibit the proliferation and differentiation of murine MC3T3-E1 pre-osteoblast cells in a concentration-dependent manner, with antagonist CH223191 pretreatment restoring their differentiation potential (Yu H. et al., 2014). These studies, therefore, infer that AHR may have distinct effects in differentiation and pluripotency depending on the cell type, in a similar way to what happens in cell proliferation and migration (Pohjanvirta et al., 2012). Moreover, Hippo signaling pathway, responsible for the first fate decision establishment in morula stage mouse embryos, was also upregulated in AHR^−/−^ embryos, contributing to the differentiation of extra-embryonic tissues. In this context, AHR has a pro-differentiation role in the early mouse embryo needed to specify the different cell fates (Nacarino-Palma et al., 2021a).

### Aryl Hydrocarbon Receptor Role in Chromatin Dynamics

Interestingly, the regulation of cell fate and differentiation is also related with transcriptional regulation by retrotransposable elements (Mulero-Navarro and Fernandez-Salguero, 2016). Being part of the family of mobile elements, retrotransposons contains the SINE (Short Interspersed Nuclear Elements), LINE (Long Interspersed Nuclear Elements) and LTR (Long Terminal Repeat) subtypes (Batzer and Deininger, 2002; Deininger et al., 2003). Although these mobile elements were described several decades ago (Vasicek et al., 1997; Kondo-Iida et al., 1999), their role in development and pathophysiology has only become known in the last decade, with AHR showing a strong role in their regulation (Roman et al., 2008; Gogvadze and Buzdin, 2009; Román et al., 2011; Morales-Hernández et al., 2016; González-Rico et al., 2020).

In recent years, studies on the position that regulatory elements occupy throughout the genome (promoters, repressor elements, enhancers, and insulators, among others) have acquired special importance. Therefore, chromatin is not positioned randomly within the nucleus. Chromosomes can organize themselves into topologically associated domains, with a size of mega bases, called topological associated domains (TADs). Long-range interactions between regulatory and promoter elements in these domains is high (Dixon et al., 2012). Therefore, the relationship between the position of a gene in the context of the nuclear chromatin structure and its level of gene expression is widely accepted (Gibcus and Dekker, 2013). The transcriptional repressor CTCF (11-zinc finger protein or CCCTC binding factor) actively participates in these long-range interactions. Originally described as a c-Myc repressor in chicken (Filippova et al., 1996), it was later found to possess enhancer-blocking activity at said locus (Recillas-Targa et al., 2002). Considered the insulator element by excellence, CTCF most known function is to attract loci that are distant within the same chromosome and even between different chromosomes (Phillips-Cremins and Corces, 2013). It has been described that the cooperation between CTCF and AHR is involved in the insulating activity of the retrotransposon of the SINE-B1 family known as B1X35S, which represses the expression of target genes such as Rtl1, Dad1 and Tbc1d1 (Román et al., 2011). Interestingly, B1X35S has functional XRE and E-box sites to which AHR and Slug / SNAI2 bind and whose mutation blocks its isolating activity (Roman et al., 2008). Furthermore, while the basal transcription of the B1X35S element is dependent on RNA polymerase III (RNA pol III), its transcription is dependent on the binding of AHR to its XRE site involves the recruitment of RNA polymerase II (RNA pol II) and the release of RNA pol III (Román et al., 2011). Regarding that, other studies have shown that AHR was required for retinoic acid (RA)-mediated differentiation of N-TERA2 cells, specifically RA-induced differentiation promoted AHR binding to Alu retrotransposons flanking pluripotency genes NANOG and OCT4. Notably, Alu-generated transcripts in differentiated cells were able to repress NANOG and OCT4 expression by a mechanism involving the miRNA machinery. Interestingly, such repressive mechanism appears to be mediated by non-coding RNA transcripts produced by RNA pol III from the Alu elements following AHR binding (Morales-Hernández et al., 2016). On top of that, it was also unveiled the existence of a complex regulatory network of proteins such as PRMT1 and CHAF1B involved in chromatin architecture and assembly, epigenetics and chromatin dynamics that control the formation of a chromatin loop between two Alu retrotransposons flanking the NANOG loci. As a consequence, NANOG expression can be downregulated during differentiation process in human teratocarcinoma N-TERA2 cell line in an AHR-dependent manner (González-Rico et al., 2020).

On the other hand, regions of DNA located in the inter-nucleosomal spaces have been described that present high accessibility for the binding of transcription factors, which are used as platforms for the binding of proteins responsible for preventing chromatin relaxation. In fact, CTCF, which has binding sites throughout the genome, could contribute to establishing heterochromatin barriers capable of modulating gene expression at the genomic level (genome-wide) depending on cell types and specific physiological context (Fu et al., 2008; González-Rico et al., 2020). Therefore, it is worth highlighting the recent interest in studying the possible relationship between chromatin accessibility and the implication of AHR over the regulation of gene expression, based on the presence of binding sites for enhancers and insulators.

## Cell Reprogramming: A New Path for Aryl Hydrocarbon Receptor

Cell reprogramming involves genetically reversing cell identity so that a differentiated cell acquires pluripotent characteristics. Such identity is conferred by its phenotype, lineage and state, and its underlying molecular regulation could provide the possibility of cell fate understanding and manipulation (Morris, 2019). Since the first isolation of embryonic stem cells (ESCs), many efforts have been made to understand and characterize the mechanisms involved in the maintenance of pluripotency.

Cell differentiation was once thought to be an irreversible process, until an initial work provided the first evidence that certain factors can erase cell identity (Gurdon et al., 1958). Decades later, it was revealed that the transcription factors Oct4, Sox2, Klf4 and c-Myc (OSKM) were enough to reprogram a terminally differentiated cell into a pluripotent cell, known as an induced pluripotent stem cell (iPSC) (Takahashi and Yamanaka, 2006). Several studies combining these factors determined that iPSCs were functionally identical to ESCs, therefore, they could be differentiated into adult cells of any lineage (Dimos et al., 2008; Chambers et al., 2009; Karumbayaram et al., 2009). The core transcriptional network OCT4, SOX2 and NANOG is also responsible for regulating the maintenance of pluripotency in ESCs (Jaenisch and Young, 2008; Young, 2011).

The most common criteria to determine the efficiency of iPSC reprogramming are both the number of new colonies with typical stem cell morphology (Cho et al., 2010; Jia et al., 2010) and the number of clones expressing alkaline phosphatase (Fusaki et al., 2009; Kim et al., 2009). In this way, a high efficiency is caused by several factors such as cell senescence and proliferation status (Zhao et al., 2008; Hanna et al., 2009; Utikal et al., 2009), MET-related factors (Samavarchi-Tehrani et al., 2010), expression of the NANOG transcription factor (Takahashi and Yamanaka, 2006; Silva et al., 2009; Theunissen et al., 2011), MAPK and GSK3 pathway inhibitors (Ying et al., 2008) and methylation inhibitors (Mikkelsen et al., 2008; Theunissen et al., 2011).

Lately, there are a growing number of studies who achieve cell reprogramming with several pathways with both *in vitro* and *in vivo* models. Regarding *in vitro* ones, the adult cell can revert to a pluripotent state and then differentiate into the desired cell type (Brambrink et al., 2008; Stadtfeld et al., 2008). Another option is to express specific factors to directly modify a cell with a different identity (Aydin and Mazzoni, 2019), a method known as lineage reprogramming (Jopling et al., 2011). *In vivo*, several reprogrammable mouse models expressing Yamanaka factors (OSKM) after induction with doxycycline treatment have been established (Abad et al., 2013; Ohnishi et al., 2014; Ocampo et al., 2016). Therefore, cell reprogramming is an emerging alternative to promote tissue regeneration and self-repair in the follow-up of diseases (Sánchez Alvarado and Yamanaka, 2014; Jessen et al., 2015; Passier et al., 2016; Takahashi and Yamanaka, 2016).

Surprisingly, cell reprogramming appears closely linked to senescence, a seemingly opposite cellular state that represents a hallmark of aging in response to various stress stimuli (López-Otín et al., 2013; Chiche et al., 2020). Recent observations support that tissue injury can induce senescence and activates signaling pathways that control reprogramming, thus highlighting the functional association of both processes (Mosteiro et al., 2016b; Chiche et al., 2017; Mosteiro et al., 2018). Therefore, the relationship between senescence and reprogramming has become a new trend to explore. The opposing effects of reprogramming factors on the senescence response (between complete reprogramming and partial reprogramming) could be a consequence of their level of induction and duration (Chiche et al., 2020). This leads to a challenging understanding of the reprogramming process and its potential clinical research application.

One of the most intriguing features of AHR is that its role in both oncogenesis and stemness is conditioned by the cell type, acting as a tumor suppressor or as an oncogene upon specific cell types, tissues or organs (Marlowe and Puga, 2005; Barouki et al., 2007). Recent studies have identified reprogramming and pluripotency factors (OSKM) as involved in the progression of different tumor types (Yin et al., 2015; Kuo et al., 2016; Zhou et al., 2016). In turn, AHR constitutively represses the expression of the c-Myc oncogene in mammary gland tumor lines (Yang et al., 2005). Also, AHR induces human teratocarcinoma cells differentiation by repressing NANOG and OCT4 expression through an Alu retrotransposon mediated mechanism (González-Rico et al., 2020), suggesting that AHR may activate a mechanism that controls the expression of pluripotency genes in both pluripotent and differentiation states. Other studies have shown that the pro-tumor and pro-metastatic activity observed in melanoma cells upon AHR absence is associated with the activation of the pluripotency inducer SOX2 and the aldehyde dehydrogenase enzyme IAI (ALDH1A1) (Contador-Troca et al., 2015). Consequently, such deregulation of AHR activity has important implications in cancer.

These and other evidence suggest that AHR could play a central role in the regulation of pluripotency, and thus reprogramming. Potential mechanisms through which AHR modulates pluripotency are regulation of cell cycle, epigenetic regulation through DNA methylation and interplay between AHR and pluripotency factors in stem cells (Ko and Puga, 2017).

Being a key factor in differentiation, AHR has a relevant implication in stemness maintenance. Its expression in embryonic stem cells is transcriptionally repressed by signaling pathways involving the pluripotency factors Oct4, Nanog, Sox2 and Polycomb proteins (Ko et al., 2014). Thus, the anti-allergic drug tranilast can reverse differentiation and promote reprogramming of mouse embryonic fibroblasts to induced pluripotent stem cells (iPSCs) by modulation of the microRNA miR-302 through AHR (Hu et al., 2013). Furthermore, it has been suggested that AHR repression is necessary to prevent premature loss of pluripotency and to maintain mitotic progression of embryonic stem cells (Ko, Fan, de Gannes, et al., 2016). Therefore, although AHR expression in embryonic stem cells is under the control of the pluripotency factor network, increased AHR expression is likely to counteract the maintenance of pluripotency and induce exit from the pluripotent state.

Recently, it has also been described that AHR deficiency promotes complete tissue repair in the lung after acute toxicity, implicating the expansion of stem cells expressing reprogramming and pluripotency factors OCT4, NANOG and CK14 (Morales-Hernández et al., 2017). Not limited to this tissue, it has been additionally reported an earlier and more efficient liver regeneration, resulting in a response of increased proliferative potential and expansion of cells expressing OCT4, NANOG and TBX3 factors (Moreno-Marín et al., 2017). The use of experimental models, in which AHR expression has been interfered with, shows a more undifferentiated phenotype and ultimately a more pluripotent basal state, which has consequently, among others yet to be identified, a more effective regenerative capacity (Morales-Hernández et al., 2017; Moreno-Marín et al., 2017). Such enhanced regenerative capacity also appears when major lung stem cells responsible for regeneration and repair after injury, including type-II alveolar cells and Clara cells, are amplified in K-Ras^G12D/+^; AHR ^−/−^ NSCLC lesions (Nacarino-Palma et al., 2021b). This links to the opportunity offered by cellular reprogramming in the research of the rejuvenation process (Mahmoudi and Brunet, 2012; Mahmoudi et al., 2019), highlighting its relevance in the use of cell reprogramming in iPSC-based regenerative therapies.

In conclusion, AHR presents a key involvement in numerous critical signaling pathways for the maintenance of cellular homeostasis, which makes its role characterization in them a must.

## Future Directions

Cellular differentiation was described decades ago and has long been considered responsible for the irreversible loss of proliferative capacity and the acquisition of a target defined and terminal cell. However, seminal findings in recent years have surprisingly revealed that a terminally differentiated cell can reprogram their gene expression pattern and dedifferentiate into a pluripotent state (induced Pluripotent Stem Cell, iPS) from which a cell type different from that of departure. The intensive research on AHR in recent years has led to the conclusion that, in addition to its functions in detoxification, this receptor exerts physiological and homeostatic functions in different tissues and organs including liver, skin, heart and immune system. A notable property of AHR is that its functions can be influenced by the phenotype of the target cell. Thus, it can promote or inhibit cell proliferation and tumor progression by acting as an oncogene or as a tumor suppressor. Overall, all these new findings suggest that dysregulation of AHR may have a causal role contributing to tumor progression and spread. For that reasons, one plausible hypothesis is that the AHR has a regulatory function in the reprogramming-senescence axis that ultimately impacts tissue regeneration. AHR would then serve as limiting factor to control the extent of tissue reprogramming and repair as well as the appearance of senescent cells in response to either toxic injury or tumorigenesis. Consequently, AHR deficiency may deregulate the reprogramming-senescence balance that, on the one hand improves tissue regeneration while, on the other, exacerbates tumor progression. This could be related with the fact that AHR is relevant in controlling the reprogramming-senescence balance that likely underlines organ regeneration. Interestingly, senescence is closely related to reprogramming as an increasingly number of reports are revealing, including the increase in senescence in reprogrammed tumors of the pancreas (Abad et al., 2013).

**FIGURE 2:**
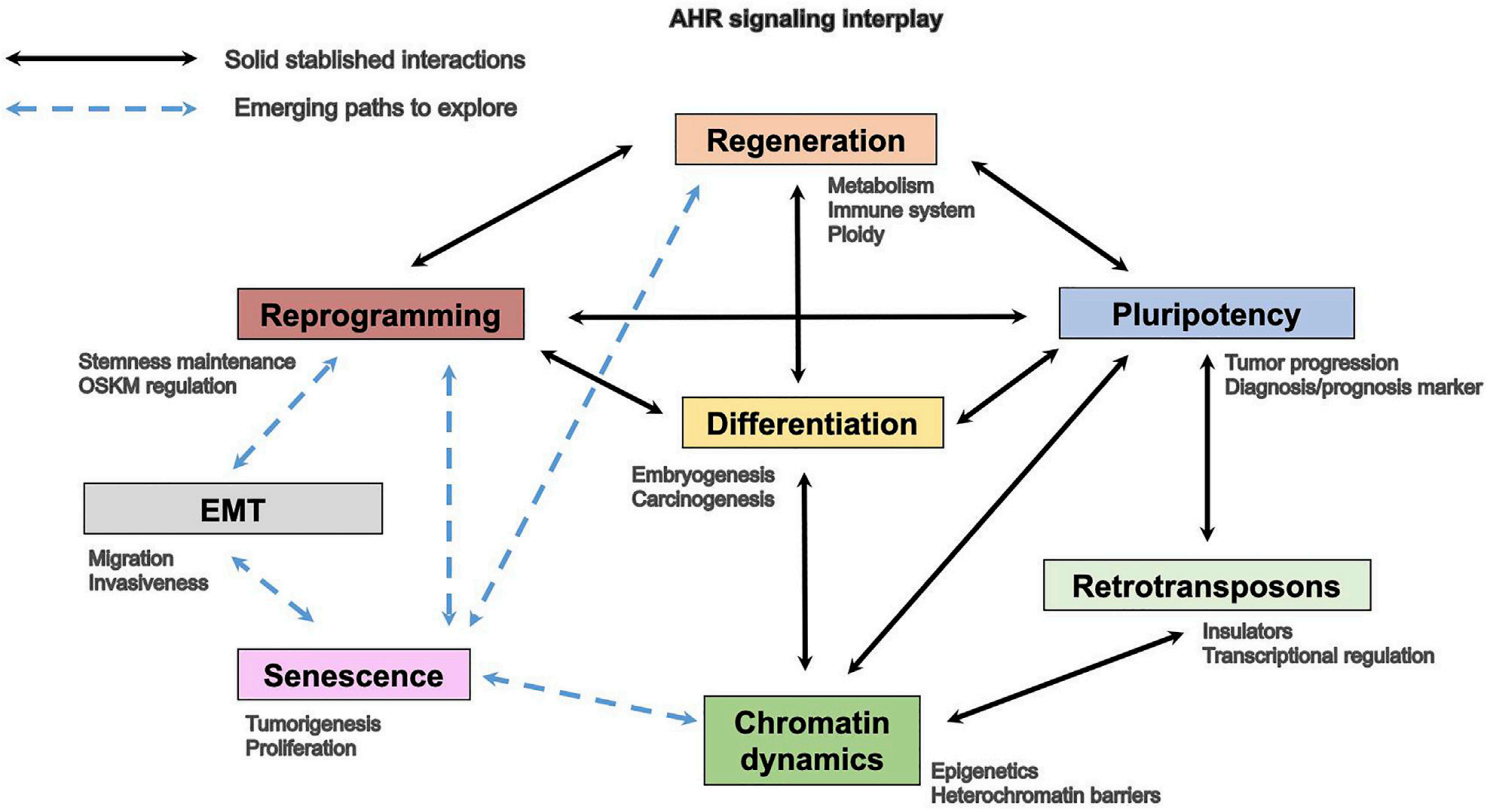
AHR involvement in homeostatic and cancer processes.

Recent investigation indicates the existence of a link between those processes in normal development and in the cell response against pathology or injury. The fact that senescence has emerged as a cell state that probably has a major impact in tissue homeostasis, therefore having functions beyond aging, opens new scientific views particularly with respect to its correlation with undifferentiation and reprogramming. It is still most interesting that recent studies suggest that, in fact, senescence is a determining factor in tissue repair and studies are ongoing trying to develop novel therapeutic tools based on selenolytic molecules able to specifically control the expansion of these cells. Therefore, there is an increasing interest in identifying novel molecular intermediates with causal roles in the control of the reprogramming-senescence-regeneration axis. Understanding the signaling pathways controlling cellular and molecular mechanisms which undergo organ differentiation, tissue repair, cell reprograming, and aging will lead the way in future studies, with AHR earning a pivotal role (Figure 2).
